# The Chemokine Fractalkine Can Activate Integrins without CX3CR1 through Direct Binding to a Ligand-Binding Site Distinct from the Classical RGD-Binding Site

**DOI:** 10.1371/journal.pone.0096372

**Published:** 2014-05-02

**Authors:** Masaaki Fujita, Yoko K. Takada, Yoshikazu Takada

**Affiliations:** Department of Dermatology, Biochemistry and Molecular Medicine, UC Davis School of Medicine, Sacramento, California, United States of America; Lerner Research Institute, United States of America

## Abstract

The chemokine domain of fractalkine (FKN-CD) binds to the classical RGD-binding site of αvβ3 and that the resulting ternary complex formation (integrin-FKN-CX3CR1) is critical for CX3CR1 signaling and FKN-induced integrin activation. However, only certain cell types express CX3CR1. Here we studied if FKN-CD can activate integrins in the absence of CX3CR1. We describe that WT FKN-CD activated recombinant soluble αvβ3 in cell-free conditions, but the integrin-binding defective mutant of FKN-CD (K36E/R37E) did not. This suggests that FKN-CD can activate αvβ3 in the absence of CX3CR1 through the direct binding of FKN-CD to αvβ3. WT FKN-CD activated αvβ3 on CX3CR1-negative cells (K562 and CHO) but K36E/R37E did not, suggesting that FKN-CD can activate integrin at the cellular levels in a manner similar to that in cell-free conditions. We hypothesized that FKN-CD enhances ligand binding to the classical RGD-binding site (site 1) through binding to a second binding site (site 2) that is distinct from site 1 in αvβ3. To identify the possible second FKN-CD binding site we performed docking simulation of αvβ3-FKN-CD interaction using αvβ3 with a closed inactive conformation as a target. The simulation predicted a potential FKN-CD-binding site in inactive αvβ3 (site 2), which is located at a crevice between αv and β3 on the opposite side of site 1 in the αvβ3 headpiece. We studied if FKN-CD really binds to site 2 using a peptide that is predicted to interact with FKN-CD in site 2. Notably the peptide specifically bound to FKN-CD and effectively suppressed integrin activation by FKN-CD. This suggests that FKN-CD actually binds to site 2, and this leads to integrin activation. We obtained very similar results in α4β1 and α5β1. The FKN binding to site 2 and resulting integrin activation may be a novel mechanism of integrin activation and of FKN signaling.

## Introduction

Fractalkine (FKN, CX3CL1) is a membrane-bound chemokine of the CX3C family [1], [2]. FKN is expressed on the cell surface of IL-1- and TNFα-activated endothelium as a membrane-bound form [2]. FKN has an N-terminal chemokine domain (residues 1–76) [3]. FKN is cleaved by metalloproteinases ADAM-10 (A Disintegrin And Metalloprotease 10) and ADAM-17 and soluble FKN is released [4]–[6]. FKN's highly selective receptor CX3CR1 (a G-protein coupled receptor, GPCR) is expressed in monocytes, T cell, NK cells, and neuron [7]–[13]. Interaction between membrane-bound FKN and CX3CR1 promotes leukocyte adhesion to endothelium [7], [14], [15].

Integrins are a family of cell adhesion receptors that recognize extracellular matrix ligands and cell surface ligands [16]. Activated integrins support both cell migration and adhesion in a cation-dependent manner. Upon activation, integrins undergo a series of conformational changes that result in increased binding affinity for their respective ligands [17]. FKN enhances cell adhesion through integrin activation that triggers arrest and firm adhesion. It has been well established that FKN-mediated integrin activation is typically mediated by CX3CR1 engagement [15], [18]–[24].

We recently discovered that the chemokine domain of FKN (FKN-CD) binds to integrins α4β1 and αvβ3 [25]. The affinity of FKN-CD binding to αvβ3 is extremely high as an integrin ligand (KD = 3.0×10^−10^ M in Mn^2+^). FKN-CD binds to the ligand-binding site common to other known integrin ligands (classical RGD-binding site). The integrin-binding defective FKN-CD mutant (the Lys36 to Glu/Arg37 to Glu (K36E/R37E) mutant) is defective in FKN signaling, while it still binds to CX3CR1. CX3CR1, FKN-CD, and integrin make a ternary complex through the direct integrin binding to FKN-CD. We propose a model in which FKN on endothelial cells binds to leukocytes through CX3CR1 and integrins (αvβ3 and α4β1), and in which integrins are directly involved in FKN signaling and leukocyte trafficking through binding to FKN-CD. We demonstrated that K36E/R37E suppressed CX3CR1 signaling (integrin activation) in a concentration-dependent manner [25], suggesting that K36E/R37E is a dominant-negative antagonist of CX3CR1.

The expression of CX3CR1 is limited to certain cell types. In the present study, we studied if FKN-CD can activate integrins in the absence of CX3CR1. We describe that FKN-CD can activate αvβ3 in the absence of CX3CR1, but that this activation requires the direct binding of FKN-CD to αvβ3. We hypothesized that FKN-CD enhances ligand binding to the classical RGD-binding site (site 1) through binding to a second binding site (site 2) that is distinct from site 1 in αvβ3. We identified a potential FKN-CD-binding site (site 2), which is located at a crevice between αv and β3 on the opposite side of site 1 in the αvβ3 headpiece. We provide evidence that FKN-CD actually binds to site 2, and this leads to integrin activation. The FKN binding to site 2 and resulting integrin activation may be a novel mechanism of integrin activation and of FKN signaling.

## Materials and Methods

### Materials

K562 erythroleukemia cells, U937 monocytic cells, and Chinese hamster ovary (CHO) cells were obtained from the American Type Culture Collection. K562 cells expressing human integrin αvβ3 (αvβ3-K562) [26] were provided by Eric Brown (University of California, San Francisco, CA). K562 cells expressing human integrin α4 (α4-K562), CHO cells expressing human integrin β3 (β3-CHO) or integrin α4 (α4-CHO) were described [27]. Recombinant soluble αvβ3 was synthesized in CHO-K1 cells using the soluble αv and β3 expression constructs and purified by Ni-NTA affinity chromatography as described [28]. The disintegrin domain of ADAM-15 (GST fusion protein) was synthesized as described [29]. Fibrinogen γ-chain C-terminal domain that lacks residues 400–411 (γC399tr) was synthesized as described [30]. GST-fusion proteins of fibronectin type III domains 8–11 (FN8-11), and fibronectin H120 fragment (FN-H120) were described [25]. We confirmed that these ligands are properly folded, since heat treatment of the ligands effectively suppressed their binding to integrins upon stimulation by FKN-CD in the binding assay (see below)(Figure S1). Vascular cell adhesion molecule-1 (VCAM-1) was provided by Novartis. Anti-CX3CR1 antibody was purchased from AbD Serotec. Anti-human β3 mAb AV10 was provided by B. Felding-Habermann (The Scripps Research Institute, La Jolla, CA). HRP-conjugated anti-His tag antibody was purchased from Qiagen (Valencia, CA).

### Synthesis of FKN-CD

Recombinant FKN-CD (WT and K36E/R37E) were synthesized as described [25] using PET28a expression vector. The proteins were synthesized in BL21 induced by isopropyl β-D-thiogalactoside as insoluble proteins. The proteins were solubilized in 8 M urea, purified by Ni-NTA affinity chromatography under denatured conditions, and refolded as previously described [31]. The refolded proteins were >90% homogeneous upon SDS-PAGE.

### Synthesis of site 2 peptides

We introduced 6His tag to the BamHI site of pGEX-2T using 5′-GATCTCATCATCACCATCACCATG-3′ and 5′-GATCCATGGTGATGGTGATGATGA-3′ (resulting vector is designated pGEX-2T6His). We synthesized GST fusion protein of site 2 peptide (QPNDGQSHVGSDNHYSASTTM, residues 267–287 of β3, C273 is changed to S) and a scrambled site 2 peptide (VHDSHYSGQGAMSDNTNSPQT) by subcloning oligonucleotides that encodes these sequences into the BamHI/EcoRI site of pGEX-2T6His. We synthesized the proteins in BL21 cells and purified using glutathione-Sepharose affinity chromatography. The corresponding β1, β2, and β4 peptides were generated as described above.

### Binding of soluble αvβ3 to γC399tr or ADAM-15

ELISA-type binding assays were performed as described previously [25]. Briefly, wells of 96-well Immulon 2 microtiter plates (Dynatech Laboratories, Chantilly, VA) were coated with 100 µl PBS containing γC399tr or ADAM-15 for 2 h at 37°C. Remaining protein binding sites were blocked by incubating with PBS/0.1% BSA for 30 min at room temperature. After washing with PBS, soluble recombinant αvβ3 (5 µg/ml) in the presence or absence of FKN-CD (WT or K36E/R37E) was added to the wells and incubated in HEPES-Tyrodes buffer (10 mM HEPES, 150 mM NaCl, 12 mM NaHCO_3_, 0.4 mM NaH_2_PO_4_, 2.5 mM KCl, 0.1% glucose, 0.1% BSA) with 1 mM CaCl_2_ for 2 h at room temperature. After unbound αvβ3 was removed by rinsing the wells with binding buffer, bound αvβ3 was measured using anti-integrin β3 mAb (AV-10) followed by HRP-conjugated goat anti-mouse IgG and peroxidase substrates.

### Flow cytometry

The cells were cultured to nearly confluent in RPMI 1640/10% FCS (K562) or DMEM/10% FCS (CHO cells). The cells were resuspended with RPMI 1640/0.02% BSA or DMEM/0.02% BSA and incubated for 30 min at room temperature to block remaining protein binding sites. The cells were then incubated with WT FKN-CD or K36E/R37E for 5 min at room temperature and then incubated with FITC-labeled integrin ligands (γC399tr, FN8-11, or FN-H120) for 15 min at room temperature. For blocking experiments, FKN-CD was preincubated with S2-β3 peptide for 30 min at room temperature. The cells were washed with PBS/0.02% BSA and analyzed by FACSCalibur (Becton Dickinson, Mountain View, CA).

### Binding of S2 peptide to proteins

ELISA-type binding assays were performed as described previously [25]. Briefly, wells of 96-well Immulon 2 microtiter plates (Dynatech Laboratories, Chantilly, VA) were coated with 100 µl PBS containing FKN-CD, γC399tr, FN-H120, or FN8-11 for 2 h at 37°C. GST tag was removed by thrombin digestion for FN-H120 and FN-8-11. Remaining protein binding sites were blocked by incubating with PBS/0.1% BSA for 30 min at room temperature. After washing with PBS, S2 peptides were added to the wells and incubated in PBS for 2 h at room temperature. After unbound S2 peptides were removed by rinsing the wells with PBS, bound S2 peptides (GST-tagged) were measured using HRP-conjugated anti-GST antibody and peroxidase substrates.

### GST Pull-down assays

We incubated the FKN-CD (0.01 µg, with 6His tag) with GST-tagged S2-β3 or S2-β1 peptide (5 µg) in 100 µl PBS for 2 h at 4°C and recovered proteins that bound to S2-β3 or S2-β1 peptide with glutathione-Agarose (sigma) and analyzed the bound proteins by Western blotting.

### Adhesion assays

Adhesion assays were performed as described previously [25]. Briefly, well of 96-well Immulon 2 microtiter plates were coated with 100 µl PBS containing FN8-11, ADAM-15, and VCAM-1 and were incubated for 2 h at 37°C. Remaining protein binding sites were blocked by incubating with PBS/0.1% BSA for 30 min at room temperature. After washing with PBS, K562, αvβ3-K562, and α4-K562 cells in 100 µl RPMI 1640 were added to the wells and incubated at 37°C for 1 h. After unbound cells were removed by rinsing the wells with the medium used for adhesion assays, bound cells were quantified by measuring endogenous phosphatase activity.

### Docking simulation

Docking simulation of interaction between FKN-CD and integrin αvβ3 (closed inactive form) was performed using AutoDock3 as described [32]. In the present study we used the headpiece (residues 1–438 of αv and residues 55–432 of β3) of αvβ3 (1JV2.pdb). Cations were not present in αvβ3 during docking simulation, as in the previous studies using αvβ3 (open form, 1L5G.pdb) [25], [32].

### Other methods

Treatment differences were tested using ANOVA and a Tukey multiple comparison test to control the global type I error using Prism 5.0 (Graphpad Software).

## Results

### FKN-CD activates integrins αvβ3 in cell-free conditions

**Figure 1:**
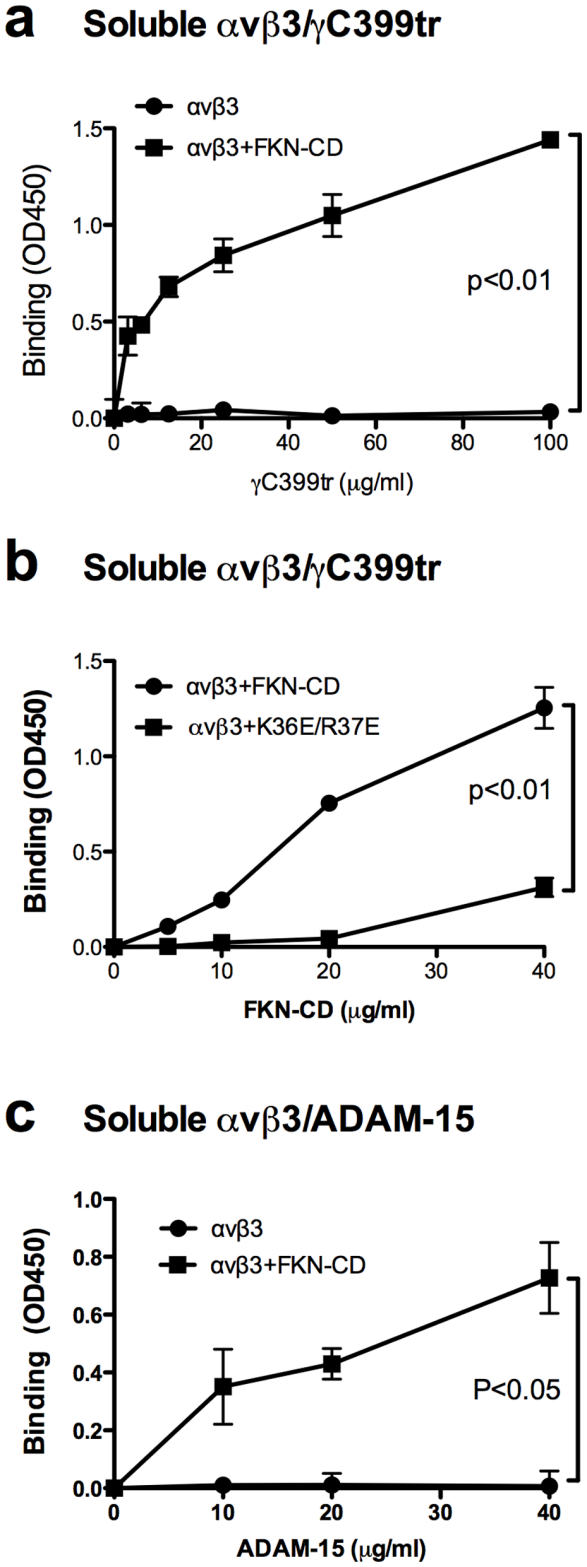
FKN-CD activates αvβ3 integrin in cell-free conditions (through direct integrin binding). a. Activation of soluble αvβ3 by FKN-CD as a function of γC399tr concentration. Binding of soluble αvβ3 (5 µg/ml) to immobilized γC399tr in the presence or absence of WT FKN-CD (40 µg/ml) was measured by ELISA. Data are shown as means ± SEM of three independent experiments. b. Activation of soluble αvβ3 by FKN-CD as a function of FKN-CD concentration. Binding of soluble αvβ3 (5 µg/ml) to immobilized γC399tr (100 µg/ml) in the presence or absence of WT FKN-CD or R36E/R37E was measured by ELISA. Data are shown as means ± SEM of three independent experiments. c. Activation of soluble αvβ3 by FKN-CD using ADAM-15 as a ligand. Experiments were done as described in (a), except that ADAM-15 and 20 µg/ml FKN-CD were used. Data are shown as means ± SEM of three independent experiments.

It has been reported that FKN rapidly enhances cell adhesion through activating integrins, which is mediated solely by CX3CR1 [7]–[9]. Is FKN totally inactive in CX3CR1-negative cell types? This question is biologically relevant since the expression of CX3CR1 is limited to certain cell types (see Introduction). We first studied if FKN-CD activates integrins in a CX3CR1-independent manner using recombinant soluble αvβ3 in cell-free conditions. WT FKN-CD markedly enhanced the binding of soluble αvβ3 (in 1 mM Ca^2+^ to keep αvβ3 inactive) to immobilized γC399tr, a specific ligand to αvβ3 [30], in a concentration-dependent manner, but K36E/R37E (the integrin-binding defective FKN-CD mutant) was defective in this function (Figures 1a and 1b). We obtained essentially the same results using another αvβ3-specific ligand, the disintegrin domain of ADAM-15 [29] (Figure 1c), suggesting that this phenomenon is not ligand specific. These results suggest that FKN-CD activates integrins without CX3CR1 in a cell-free condition and this activation requires direct integrin binding of FKN-CD.

### FKN-CD-induced integrin activation occurs in CX3CR1-negative cells

**Figure 2:**
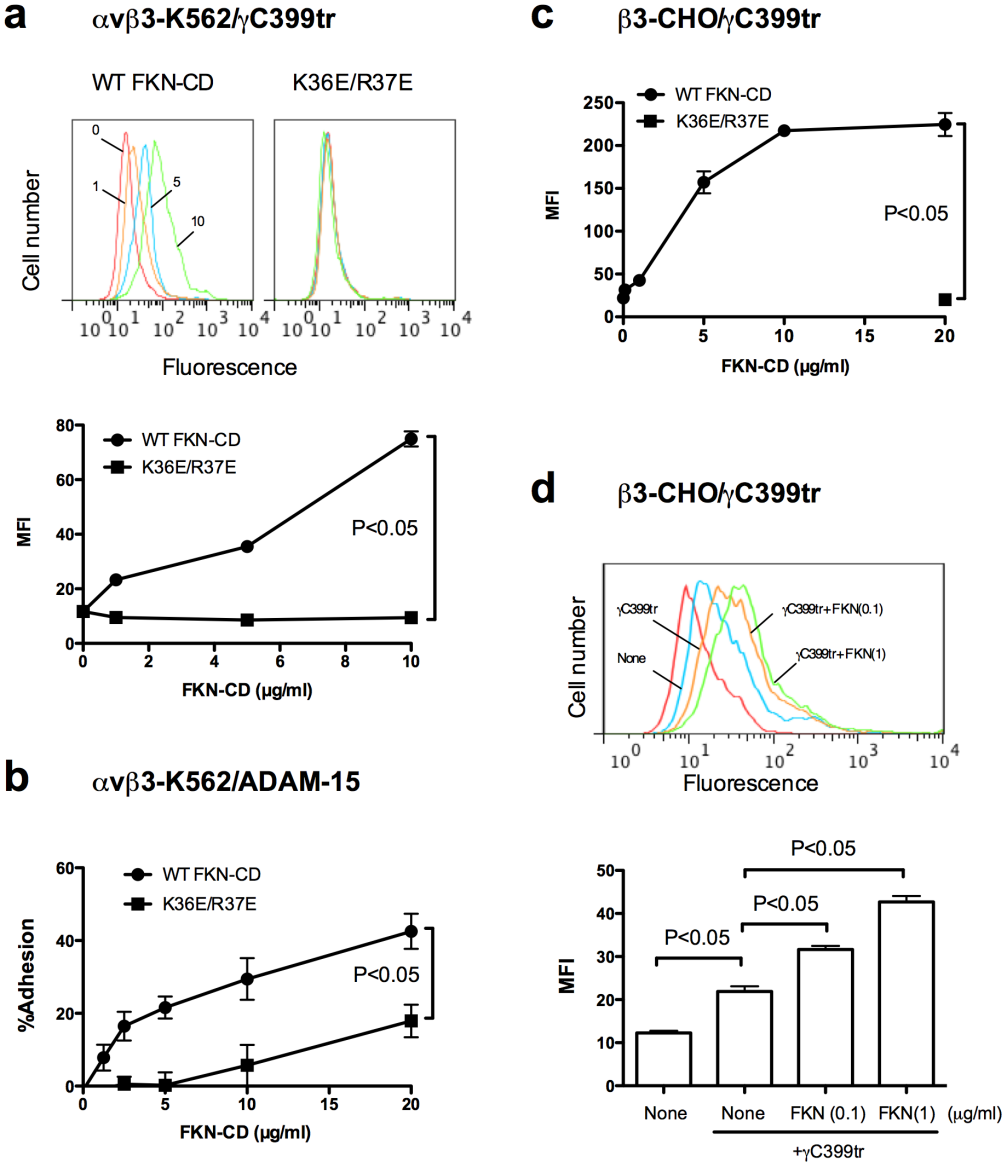
FKN-CD activates αvβ3 integrin on the surface of cells that do not express CX3CR1 (through direct integrin binding). a. Activation of αvβ3 by WT FKN-CD, but not by K36E/R37E (integrin-binding defective), in αvβ3-K562 cells (CX3CR1-negative). Cells were incubated with FITC-labeled γC399tr in the presence of WT FKN-CD or K36E/R37E. Binding of γC399tr to cells was measured by flow cytometry. Data are shown as means ± SEM of median fluorescent intensity (MFI) of three independent experiments. b. αvβ3 activation by FKN-CD as measured by adhesion to ADAM-15. Adhesion assays were performed as described in the methods. Data are shown as means ± SEM of three independent experiments. c. Activation of αvβ3 by FKN-CD in β3-CHO cells (CX3CR1-negative). Experiments were performed as described in (a) except that β3-CHO cells (CX3CR1-negative) were used instead of αvβ3-K562 cells. Data are shown as means ± SEM of MFI of three independent experiments. d. Activation of αvβ3 by FKN-CD in β3-CHO cells at low FKN-CD concentrations. Experiments were performed as described in (c) except that FKN-CD was used at 0.1 and 1 µg/ml. Data are shown as means ± SEM of MFI of three independent experiments.

We studied if FKN-CD activates αvβ3 at the cellular level in a CX3CR1-independent manner. It has been reported that K562 cells do not express detectable CX3CR1 mRNA and do not bind to soluble or membrane-bound FKN, while K562 cells that express recombinant CX3CR1 robustly bind to FKN [14]. We also confirmed that CX3CR1 is not detectable in αvβ3-K562 cells by western blotting (data not shown). We studied if FKN-CD induces αvβ3 activation using K562 cells that express recombinant αvβ3 (αvβ3-K562 cells). To reduce the basal levels of integrin activation 1 mM Ca^2+^ was included in the assay medium. The binding of FITC-labelled γC399tr was measured in flow cytometry. Interestingly, WT FKN-CD enhanced the binding of γC399tr to αvβ3-K562 in a concentration-dependent manner, but K36E/R37E did not (Figure 2a). The original observation of FKN-induced integrin activation is enhanced cell adhesion to substrate by FKN in a CX3CR1-dependent manner [7]. We found that FKN-CD can enhance cell adhesion of αvβ3-K562 cells to ADAM-15 (Figure 2b). These results suggest that FKN-CD can directly activate αvβ3 in the absence of CX3CR1 and this activation can be detected using different binding assays and different integrin ligands.

The FKN-induced αvβ3 activation in αvβ3-K562 cells may be cell-type specific. So we performed similar experiments using another CX3CR1-negative cells. It has been reported that CHO cells do not express detectable CX3CR1 mRNA and do not bind to FKN or show intracellular signals in response to FKN (Ca^2+^ mobilization, MAP kinase activation or AKT activation), while CHO cells that express recombinant CX3CR1 do [33]–[35]. We also confirmed that CX3CR1 is not detectable in β3-CHO cells by western blotting (data not shown). Using CHO cells that express recombinant αvβ3 (β3-CHO cells), we obtained the results that are very similar to those of αvβ3-K562: FKN-CD markedly enhanced the binding of γC399tr to β3-CHO cells in a concentration-dependent manner, but K36E/R37E was defective in this function (Figure 2c). These findings suggest that FKN-CD induced αvβ3 activation in a CX3CR1-independent manner is not cell-type specific. Thus there may be another mechanism of FKN-CD-induced integrin activation in addition to the well-established CX3CR1-mediated pathway (inside-out signaling).

FKN is used at 10–100 nM in other studies [15], [36], [37] (equivalent to 0.12–1.2 µg/ml FKN-CD). We found that FKN-CD at 0.1–1 µg/ml induced detectable integrin activation in β3-CHO cells in the absence of CX3CR1 in our binding assays (Figure 2d). This suggests that CX3CR1-independent integrin activation by FKN-CD also occurs at FKN levels that have been widely used.

### Docking simulation predicts that there is a second FKN-CD binding site in αvβ3

**Figure 3:**
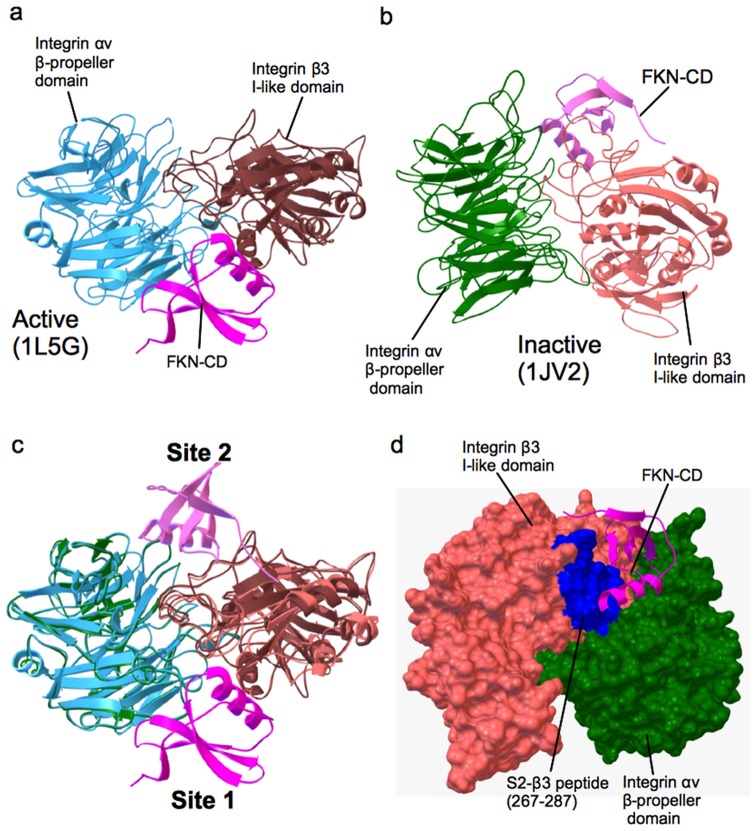
Docking simulation of FKN-CD binding to αvβ3 with an inactive conformation predicts a new ligand-binding site (site 2). a. A docking model of FKN-CD-integrin αvβ3 (active) interaction [25]. The headpiece of ligand-bound form of integrin αvβ3 (PDB code 1L5G) was used as a target. The model predicts that FKN-CD (PDB code 1F2L, red) binds to the classical RGD-binding site of the integrin αvβ3 headpiece (site 1). b. A docking model of FKN-CD-integrin αvβ3 (inactive) interaction. The headpiece of an inactive form of integrin αvβ3 (PDB code 1JV2) was used as a target. The model predicts the position of the second FKN-CD-binding site (site 2). c. Superposition of two models shows that the positions of two predicted FKN-CD binding sites are distinct. d. Position of the β3 peptide (267–287, blue) in site 2 (S2-β3). Most of amino acid residues in this peptide are predicted to interact with FKN-CD (Table 1).

We demonstrated that FKN-CD activates integrin αvβ3 in a CX3CR1-independent manner and K36E/R37E is defective in this function, suggesting that this activation involves direct αvβ3-FKN-CD interaction. If this is the case, since the classical RGD-binding site may be occupied by αvβ3 ligands (γC399tr and ADAM15), it is hypothesized that FKN-CD binds to another binding site. The crystal structure of the active ligand-bound form of αvβ3, however, contains only one RGD-containing peptide (PDB code 1L5G) [38]. In our previous study, docking simulation of interaction between FKN-CD and active αvβ3 predicts that FKN-CD binds to the classical RGD-binding site [25] (designated site 1) (Figure 3a). We suspected that the inactive form of αvβ3, in which site 1 is in a closed conformation, may have an open second ligand-binding site. We thus performed another docking simulation of FKN-CD-αvβ3 interaction using the inactive form of αvβ3 (PDB code 1JV2) as a target. The simulation identified a second FKN-CD-binding site (docking energy -24 kcal/mol) (designated site 2) (Figure 3b), which is distinct from site 1 (Figure 3c). The predicted site 2 is located at a shallow crevice between αv and β3 on the other side of site 1.

### A peptide sequence from the predicted site 2 binds to FKN-CD and suppresses integrin activation by FKN-CD

**Figure 4:**
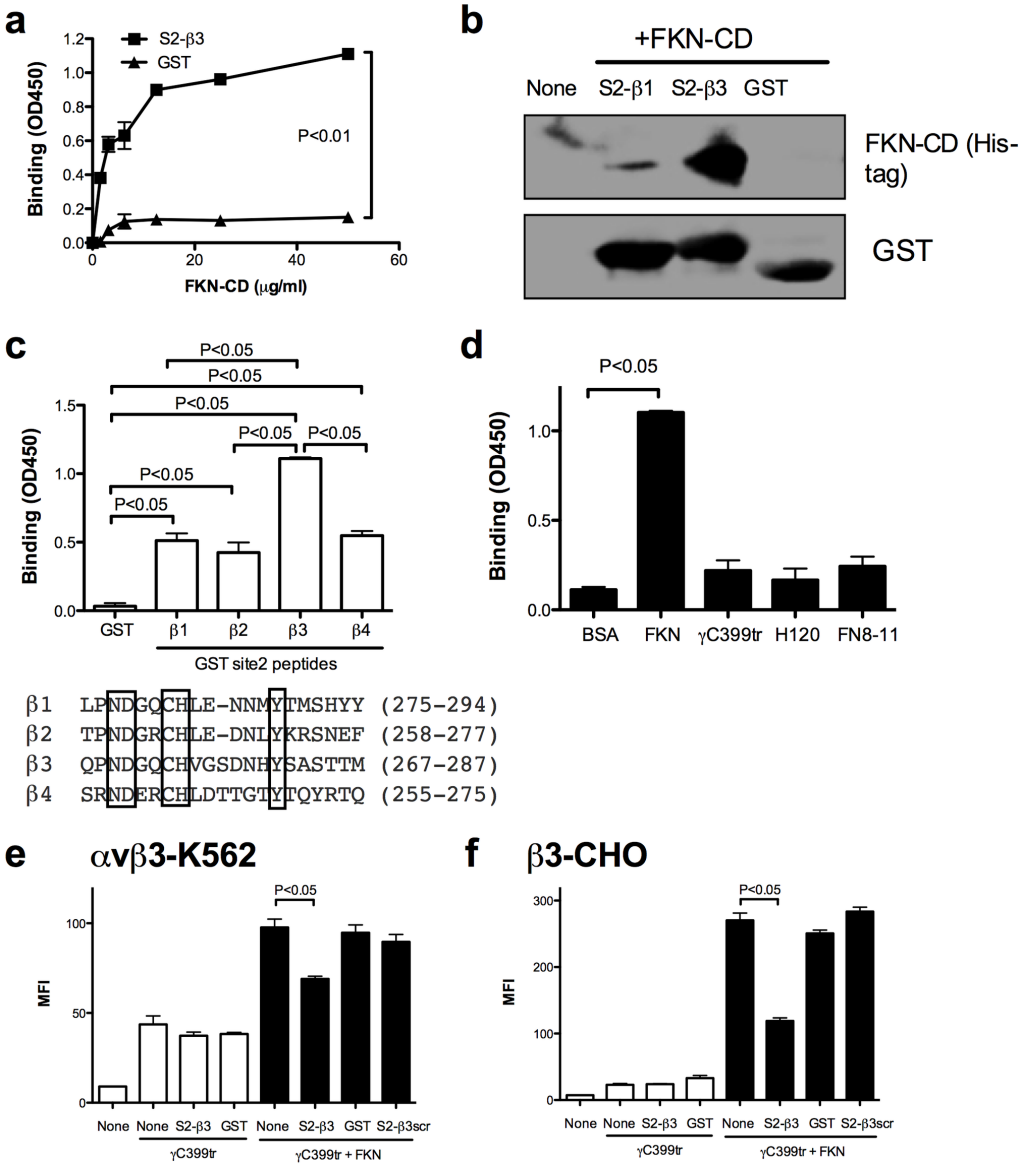
A peptide derived from the predicted site 2 of αvβ3 (S2-β3) binds to FKN-CD and suppresses CX3CR1-independent FKN-CD-induced αvβ3 activation. a. Binding of S2-β3 peptide to immobilized FKN-CD. The binding of the peptide to immobilized FKN-CD was measured by ELISA. Data are shown as means ± SEM of three independent experiments. b. Pull-down assays. FKN-CD (with 6His tag) was incubated with S2-β3 or S2-β1 peptide (GST fusion protein) and the complexes were analyzed by Western blotting. c. Binding of site 2 peptides from different integrin β subunits (S2-β1, β2, β3, and β4) to immobilized FKN-CD. The binding of peptides to immobilized FKN-CD was measured as described in (a). Data are shown as means ± SEM of three independent experiments. d. Binding of S2-β3 peptide to FKN-CD. The binding of the peptide to immobilized FKN-CD, γC399tr, FN-H120, FN-8-11 (5 µM) was measured as described in (a). Data are shown as means ± SEM of three independent experiments. e. Effect of S2-β3 peptide on FKN-CD induced integrin activation in αvβ3-K562 cells. Cells were incubated with FITC-labeled γC399tr in the presence of FKN-CD or the mixture of FKN-CD and S2-β3 peptide. FKN-CD (20 µg/ml) were preincubated with S2-β3 (300 µg/ml) in PBS for 30 min at room temperature. Binding of FITC-labeled γC399tr to cells was measured by flow cytometry. Data are shown as means ± SEM of MFI of three independent experiments. f. Effect of S2-β3 peptide on FKN-CD induced integrin activation in β3-CHO cells. The binding of γC399tr to cells was measured as described in e). Data are shown as means ± SEM of MFI of three independent experiments.

To test if FKN-CD really interacts with the predicted site 2, we selected a peptide sequence from site 2 (residues 267–287 of β3, designated S2-β3 peptide) that is predicted to interact with FKN-CD (Figure 3d and Table 1). Notably S2-β3 peptide bound to FKN-CD in a concentration-dependent manner (Figure 4a) and pulled down FKN-CD from solution (Figure 4b), control parent GST did not bind to FKN-CD. This indicates that FKN-CD specifically interacts with the predicted site 2.

**Table 1:** Amino acid residues involved in the interaction between FKN-CD and integrin αvβ3.

FKN-CD	αv	β3
Val5, Thr6, Lys18, His26, Tyr27, Gln28, Gln29, Gln31, Ala32, Ser33, Cys34, Gly35, Arg37, Pro53, Lys54, Glu55, Gln56, Trp57, Lys59, Asp60, Ala61, Met62, Gln63, His64, Asp65, Asp66, Arg67, Gln68	Glu15, Gly16, Ser17, Tyr18, Pro41, Lys42, Ala43, Asn44, Val51, Glu52, His91, Ala397, Arg398, Ser399, Met400, Pro401	Pro160, Val161, Met165, Glu171, Glu174, Asn175, Leu185, Pro186, Met187, Phe188, Lys191, Lys209, Gln210, Ser211, **Gln267, Asp270, Gln272, Cys273, His274, Val275, Gly276, Ser277, Asp278, His280, Tyr281, Ser282, Ala283, Thr285, Thr286, Met287**

Amino acid residues within 0.6 nm between FKN-CD and αvβ3 were selected using pdb viewer (version 4.1). Amino acid residues in β3 site 2 peptide (S2-β3) are shown in bold.

Since FKN-CD binds to α4β1 as well [25], we expected that site 2 is present in other integrin species. We generated peptides from the β1, β2 and β4 subunits that correspond to S2-β3 peptide (designated S2-β1, β2, and β4, respectively), and compared their ability to bind to FKN-CD. We studied if these peptides bind to FKN-CD in ELISA-type binding assays. We found that these peptides bound to FKN-CD as well, while S2-β3 peptide was the most effective (Figure 4c). These findings suggest that site 2 is present in other integrins as well, and FKN-CD is likely to activate integrins other than αvβ3.

We studied the binding specificity of S2-β3 peptide to other integrin ligands used in this study in ELISA-type binding assay. S2-β3 peptide did not significantly interact with γC399tr, α5β1-specific ligand fibronectin type III repeats 8-11 (FN8-11), and α4β1-specific fibronectin fragment H120 [39] (Figure 4d). This suggests that S2-β3 peptide does not affect the binding of these integrin ligands. As another control experiment we studied if FKN-CD directly bind to these integrin ligands in ELISA-type binding assay. We did not detect significant binding of FKN-CD to these integrin ligands (data not shown), suggesting that FKN-CD does not direct bind to these ligands.

We studied if S2-β3 peptide affects FKN-CD-induced activation of αvβ3 by measuring the binding of labeled γC399tr in the presence of FKN-CD in CX3CR1-negative cells. To show more specificity of S2-β3 peptide, we generated scrambled S2-β3 peptide (S2-β3scr peptide). Notably, S2-β3 peptide suppressed the γC399tr binding to αvβ3 increased by FKN-CD in αvβ3-K562 cells (Figure 4e) and β3-CHO cells (Figure 4f), but GST or S2-β3scr peptide did not. These results suggest that FKN-CD binding to site 2 is involved in FKN-CD-induced CX3CR1-independent αvβ3 activation.

### FKN-CD activates integrins α4β1 and α5β1 through direct binding to site 2

**Figure 5:**
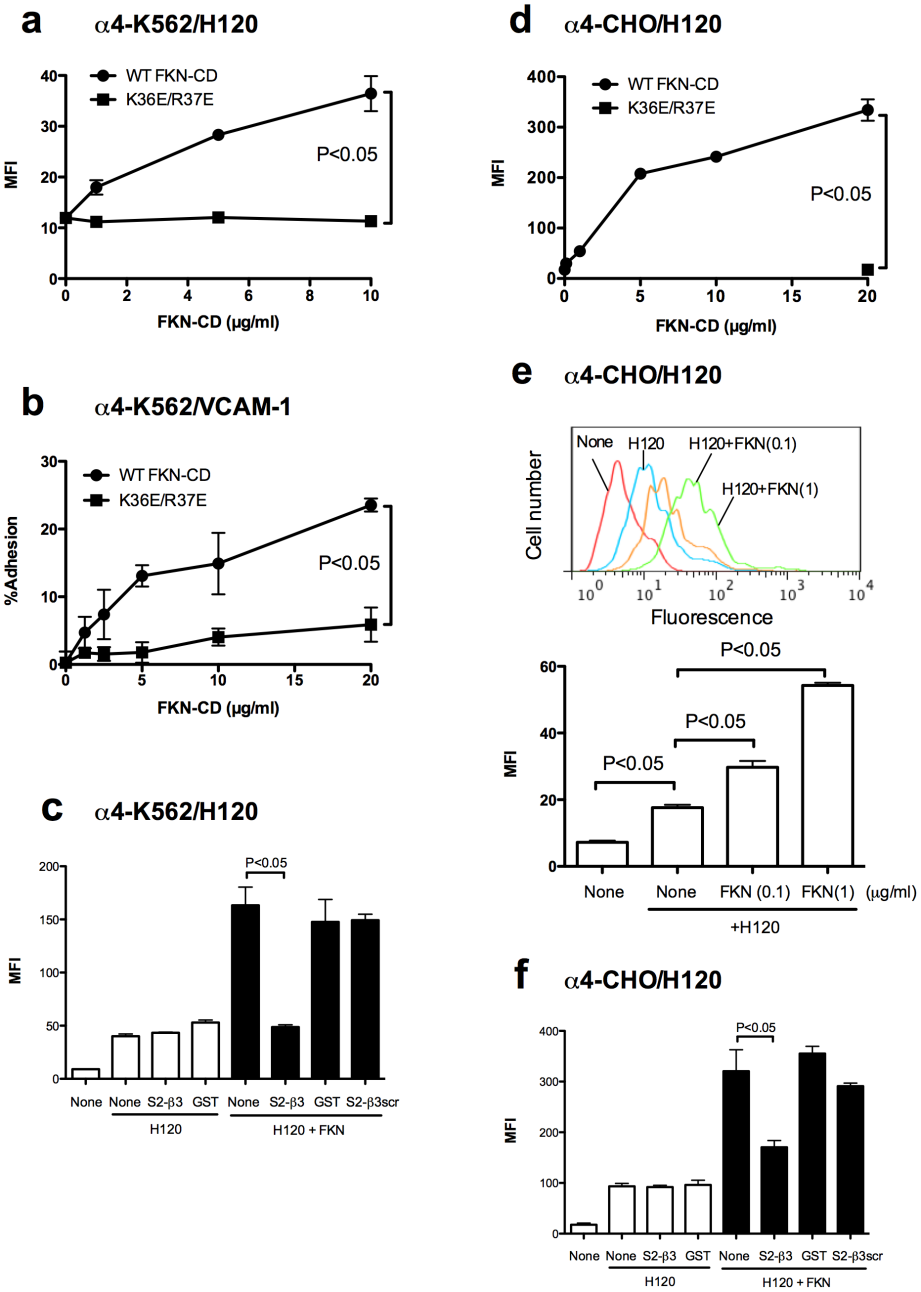
FKN-CD activates α4β1 integrin in a CX3CR1-independent manner through the binding to site 2. a. Activation of α4β1 by FKN-CD in α4-K562 cells (CX3CR1-negative). The binding of FITC-labeled H120 (specific ligand to α4β1) to cells was measured by flow cytometry. Data are shown as means ± SEM of MFI of three independent experiments. b. Adhesion of α4-K562 cell to VCAM-1. Cell adhesion to immobilized VCAM-1 was measured as described in the methods. Data are shown as means ± SEM of three independent experiments. c. Effect of S2-β3 on FKN-CD induced α4β1 activation. α4-K562 cells were incubated with FITC-labeled H120 in the presence of FKN-CD or the mixtures of FKN-CD and S2-β3. FKN-CD (20 µg/ml) was preincubated with S2-β3 (300 µg/ml) in PBS for 30 min at room temperature. Binding of FITC-labeled H120 to cells was measured by flow cytometry. Data are shown as means ± SEM of MFI of three independent experiments. d. Activation of α4β1 by FKN-CD in α4-CHO cells (CX3CR1-negative) in a CX3CR1-independent manner. The binding of FITC-labeled H120 (specific ligand to α4β1) was measured by flow cytometry. Data are shown as means ± SEM of MFI of three independent experiments. e. Activation of α4β1 by FKN-CD in α4-CHO cells at low FKN-CD concentrations. Experiments were performed as described in (d) except that FKN-CD and K36E/R37E were used at 0.1 and 1 µg/ml. Data are shown as means ± SEM of MFI of three independent experiments. f. Effect of S2-β3 peptide on FKN-CD induced integrin activation in α4-CHO cells. Experiments were performed as descibed in c) except that α4-CHO cells were used. Data are shown as means ± SEM of MFI of three independent experiments.

**Figure 6:**
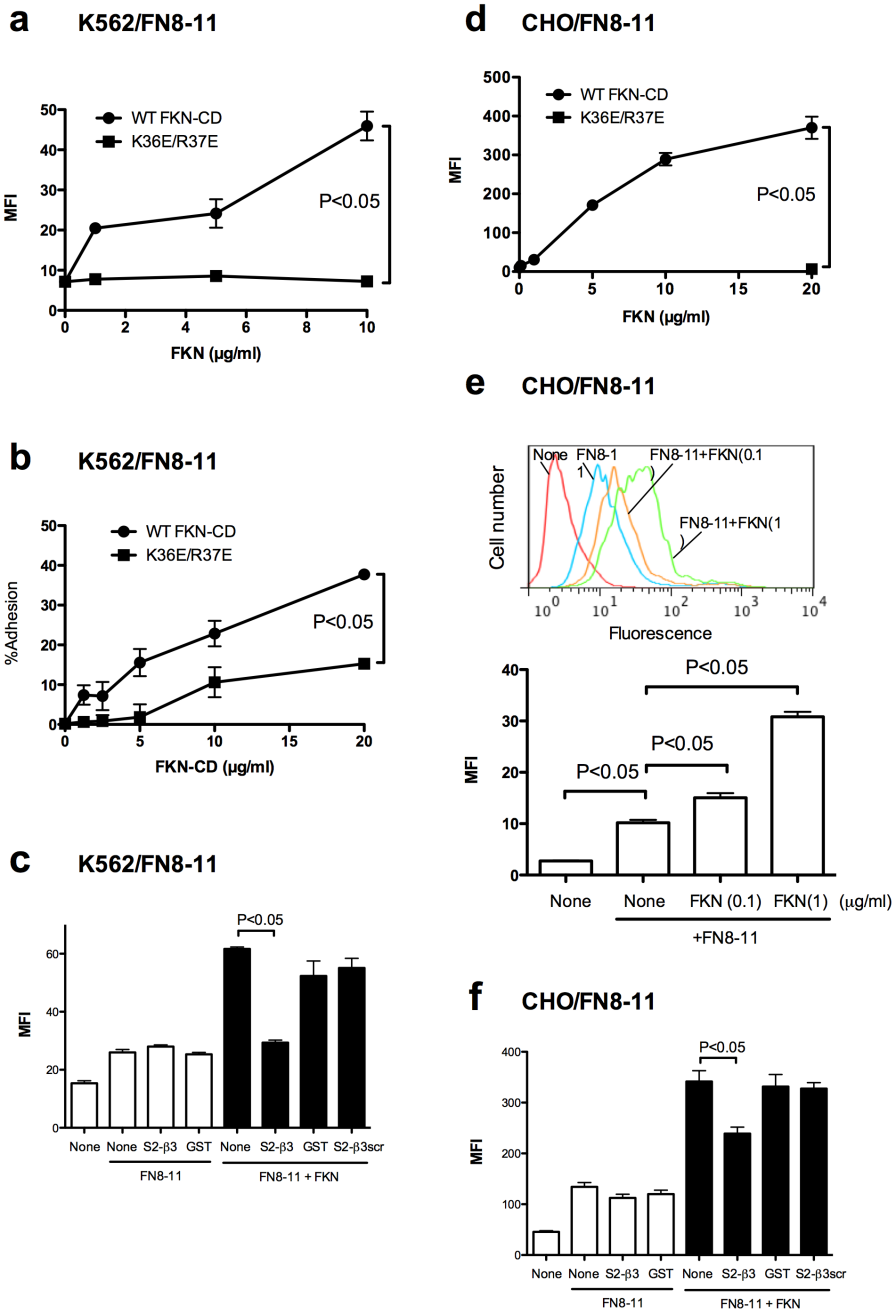
FKN-CD activates α5β1 integrin in a CX3CR1-independent manner through the binding to site 2. a. Activation of α5β1 by FKN-CD in K562 cells (CX3CR1-negative). The binding of FITC-labeled FN8-11 (specific ligand to α5β1) was measured as described in the methods. Data are shown as means ± SEM of MFI of three independent experiments. b. K562 cells adhesion to FN8-11. Cell adhesion to immobilized FN8-11 was measured as described in the methods. Data are shown as means ± SEM of three independent experiments. c. Effect of S2-β3 on FKN-CD induced integrin activation in K562 cells. Cells were incubated with FITC-labeled FN8-11 in the presence of FKN-CD or the mixtures of FKN-CD and S2-β3. FKN-CD (20 µg/ml) was preincubated with S2-β3 (300 µg/ml) in PBS for 30 min at room temperature. Binding of FITC-labeled FN8-11 to cells was measured by flow cytometry. Data are shown as means ± SEM of MFI of three independent experiments. d. Activation of α5β1 by FKN-CD in CHO cells (CX3CR1-negative) in a CX3CR1-independent manner. The binding of FITC-labeled FN8-11 (specific ligand to α5β1) was measured by flow cytometry. Data are shown as means ± SEM of MFI of three independent experiments. e. Activation of α5β1 by FKN-CD in CHO cells at low FKN-CD concentrations. Experiments were performed as described in (d) except that FKN-CD and K36E/R37E were used at 0.1 and 1 µg/ml. Data are shown as means ± SEM of MFI of three independent experiments. f. Effect of S2-β3 peptide on FKN-CD induced integrin activation in CHO cells. Experiments were performed as descibed in c) except that CHO cells were used. Data are shown as means ± SEM of MFI of three independent experiments.

We studied if FKN-CD activates integrins other than αvβ3 in a CX3CR1-independent manner. We measured the binding of FITC-labeled H120 to K562 and CHO cells that overexpress recombinant α4β1 (designated α4-K562 and α4-CHO cells, respectively) and the adhesion of α4-K562 cells to H120 (Figure 5). We also studied α5β1 activation by FKN-CD using parent K562 or CHO cells that express α5β1. We measured the binding of FITC-labeled FN8-11 and cell adhesion to FN8-11 (Figure 6). We obtained very similar results in α4β1 and α5β1 to that of αvβ3: WT FKN-CD activated α4β1 and α5β1, but K36E/R37E did not. FKN-CD at 1 µg/ml or less induced detectable α4β1 and α5β1 activation. S2-β3 peptide suppressed FKN-CD-induced α4β1 and α5β1 activation, but control GST or S2-β3scr peptide did not. These results suggest that WT FKN-CD activates integrins α4β1 and α5β1 in a CX3CR1-independent manner through binding to site 2.

## Discussion

The present study establishes that FKN-CD can activate integrins αvβ3, α4β1 and α5β1 in a CX3CR1-independent manner. We presented evidence that FKN-CD activates soluble αvβ3 in cell-free conditions and integrins on the surface of cells that do not express CX3CR1. We identified a potential mechanism of CX3CR1-independent integrin activation by FKN. Docking simulation predicted another FKN-CD binding site in the closed form of αvβ3 (site 2) that is distinct from the classical RGD-binding site (site 1). Notably, a peptide from site 2 (S2-β3) directly bound to FKN-CD and suppressed FKN-CD induced activation of integrins αvβ3, α4β1 and α5β1. These findings suggest that FKN-CD directly binds to the newly identified site 2, and this interaction potentially induces conformational changes that enhance ligand binding to site 1. We also showed that peptide sequences corresponding to S2-β3 peptide from other integrin β subunits bound to FKN-CD. This suggests that site 2 in other integrin β subunits may also be involved in FKN-CD-mediated integrin activation. These findings are consistent with the docking model. We did not detect significant binding of FKN-CD to the ligands in ELISA-type binding assays (data not shown). It is unlikely that FKN-CD affects the ligands used (γC399tr, H120, and FN8-11) and enhanced their binding to integrins. To detect the FKN-CD induced integrin activation we needed to keep integrin in an inactive state by using assay media that contain 1 mM Ca^2+^ or RPMI1640 medium that contains Ca^2+^ (approx. 0.4 mM). When αvβ3 on K562 cells is activated by 1 mM Mn^2+^, soluble γC399tr bound to αvβ3 at a maximal level without FKN-CD, and we did not detect further activation by FKN-CD or inhibition by S2-β3 peptide (data not shown). This is consistent with the idea that the direct binding of FKN-CD to site 2 activates integrins that are inactive in the physiological body fluids that contain high [Ca^2+^] (1.1–1.4 mM).

We showed that CX3CR1-independent integrin activation by FKN can be detected at 1 µg/ml FKN-CD or less in our assay system, suggesting that FKN-CD can induce CX3CR1-independent integrin activation in at FKN concentrations widely used in other studies. We recently reported that FKN-CD has KD of 10^−8^ M to integrin αvβ3 in the presence of Ca^2+^ (which reduces integrin affinity) [25]. Thus it is reasonable that FKN-CD concentration at 0.1–1 µg/ml (about 8–80 nM) may be required for FKN to bind to site 2 and activate integrins in the absence of CX3CR1. It is important to note that FKN is expressed as a membrane-bound form (e.g., of endothelial cells), unlike other soluble chemokines, and highly concentrated on the cell surface. Kinetics of interaction between membrane-bound FKN and integrins may be different from that between soluble FKN and integrins. These issues should be addressed in future studies.

We propose that integrins on CX3CR1-negative cells (such as K562) can be efficiently activated upon binding to membrane-bound FKN (on endothelial cells) in an CX3CR1-independent manner. CX3CR1 is not widely expressed (see Introduction). Interestingly only 2 out of 12 human hematopoietic cell lines tested express CX3CR1 [15]. Also, 7 breast cancer cell lines, 12 melanoma cell lines tested and their normal counterparts (mammary epithelial cells and melanocytes) do not express CX3CR1 [40]. Our results suggest that FKN may induce integrin activation (and perhaps subsequent phenotype changes) in CX3CR1-negative cells as well, which have not been recognized as target cells for FKN.

It has been well established that ligand binding to integrins induces global and/or local conformation changes in integrins. Binding of a RGD-mimetic peptide induces changes in the tertiary structure of αvβ3 [38] and αIIbβ3 [41] in the β3 I-like domain. RGD or ligand-mimetic peptides activate purified, non-activated αIIbβ3 [42] and αvβ3 [43]. This process does not require signal transduction and it appears that RGD or ligand-mimetic peptide triggers conformational changes that lead to full activation of integrins. These findings suggest that these peptides enhance integrin affinity by conformational changes in the headpiece possibly through additional ligand-binding sites in the integrin [42]. A previous study suggests that there are two RGD-binding sites in integrin αIIbβ3, and that one binding site acts as an allosteric site based on binding kinetic studies [44]. Also, another study suggests that two distinct cyclic RGD-mimetic peptides can simultaneously bind to distinct sites in αIIbβ3, and the estimated distance between two ligand-binding site is about 6.1 +/− 0.5 nm [45]. The possible allosteric ligand-binding site has not been pursued probably because the αvβ3 structure (ligand occupied, open) contains only one RGD-binding site [38]. In our docking model the distance between site 1 and site 2 is about 6 nm. Thus, the position of site 2 is consistent with the previous report. Based on previous studies it is likely that the newly identified site 2 has ligand specificity that overlaps with that of site 1, interacts with integrin ligands other than FKN-CD (e.g., RGD), and is potentially involved in integrin regulation in an allosteric mechanism. It is reasonable to assume that FKN-CD binding to site 2 induces global conformational changes in integrins. The open and closed structures of αvβ3 are superposable, while the specificity loop undergoes conformational changes (0.1 nm shift) upon RGD binding to αvβ3 [38]. It is therefore striking that docking simulation distinguished open and closed conformations of αvβ3. One possibility is that only slight changes in conformation in the headpeice (e.g., specificity loop) are involved in activation and inactivation of integrins. It would be interesting to address these questions in future studies.

Taken together the present study suggests a new mechanism of integrin activation by chemokine FKN through direct binding to integrins without inside-out signaling. This does not require CX3CR1 expression or signal transduction, and may play an important role in both CX3CR1-negative and -positive cell types. Thus the ligand-site 2 interaction may be a novel target for drug discovery and site 2 peptides may have potential as therapeutics.

## Supporting Information

Figure S1 **Heat treatment suppresses the binding of ligands to integrins.** To confirm that the recombinant integrin ligands are properly folded, we studied if heat treatment (80°C for 10 min) suppresses the binding functions of the proteins. Cells were incubated with FITC-labeled ligands (heat-treated or non-treated) in the presence or absence of WT FKN-CD. Binding of FITC-labeled ligands to cells was measured by flow cytometry. Data are shown as means ± SEM of MFI of three independent experiments. The data suggest that heat treatment significantly suppresses the FKN-induced binding of the ligands to integrins, indicating that the ligands used in this study are properly folded for integrin binding.
(TIFF)
