# Supplementary figures and images for: The Chemokine Fractalkine Can Activate Integrins without CX3CR1 through Direct Binding to a Ligand-Binding Site Distinct from the Classical RGD-Binding Site
Source: PLoS One. 2014 May 2;9(5):e96372. doi: 10.1371/journal.pone.0096372 (PMC4008574; doi:10.1371/journal.pone.0096372)

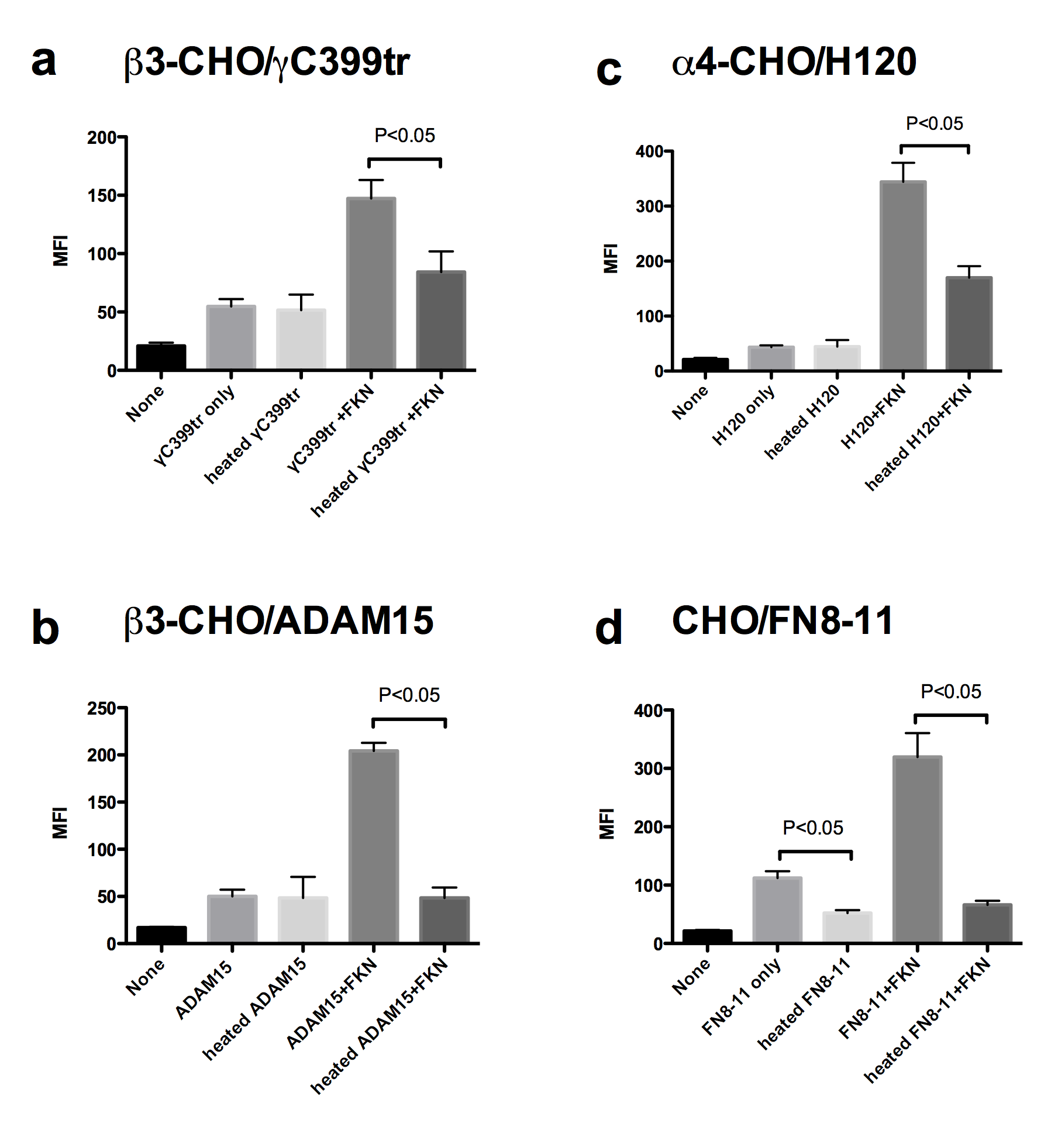

Supplement: Figure S1 — Heat treatment suppresses the binding of ligands to integrins. To confirm that the recombinant integrin ligands are properly folded, we studied if heat treatment (80°C for 10 min) suppresses the binding functions of the proteins. Cells were incubated with FITC-labeled ligands (heat-treated or non-treated) in the presence or absence of WT FKN-CD. Binding of FITC-labeled ligands to cells was measured by flow cytometry. Data are shown as means ± SEM of MFI of three independent experiments. The data suggest that heat treatment significantly suppresses the FKN-induced binding of the ligands to integrins, indicating that the ligands used in this study are properly folded for integrin binding. (TIFF) [file pone.0096372.s001.tif]
